# Tumour inoculation site-dependent induction of cachexia in mice bearing colon 26 carcinoma

**DOI:** 10.1038/sj.bjc.6690123

**Published:** 1999-02

**Authors:** T Matsumoto*, K Fujimoto-Ouchi, S Tamura, Y Tanaka, H Ishitsuka

**Affiliations:** Cytostatics Research Group, Nippon Roche Research Center, 200 Kajiwara, Kamakura, Kanagawa, 247-8530, Japan

**Keywords:** colon 26, cachexia, IL-6, inoculation site, malignancy

## Abstract

Murine colon 26 carcinoma growing at either subcutaneous (s.c.) or intramuscular (i.m.) inoculation sites causes cachexia in mice. Such animals show extensive loss of body weight, wasting of the muscle and adipose tissues, hypoglycaemia, and hypercalcaemia, even when the tumour weight comprises only about 1.9% of carcass weight. In contrast, the same tumour when inoculated into the liver does not cause any sign of tumour-related cachexia even when the tumour becomes much larger (6.6% of carcass weight). Interleukin 6 (IL-6), a mediator associated with cachexia in this tumour model, is detected at high levels both in the tumour tissues and in the circulating blood of mice bearing colon 26 tumour at the s.c. inoculation site. In contrast, only minute levels of IL-6 are detected in the tumour grown in the liver. The colon 26 tumour grown in the liver does not lose its ability to cause cachexia, because the tumour when re-inoculated s.c. is able to cause extensive weight loss and produce IL-6 as did the original colon 26 cell line. Histological studies revealed differences in the composition of tumour tissues: the tumours grown in the subcutis consist of many polygonal tumour cells, extended-intercellular space, and high vascular density, whereas those grown in the liver consist of spindle-shaped tumour cells. Thus, the environment where tumour cells grow would be a critical factor in determining the cachectic phenotype of cancer cells, including their ability to produce IL-6. *1999 Cancer Research Campaign*


*British Journal of Cancer* (1999) **79**(5/6), 764–769
© 1999 Cancer Research Campaign
Article no. bjoc.1998.0123

### Tumour inoculation site-dependent induction of cachexia in mice bearing colon 26 carcinoma

**T Matsumoto*, K Fujimoto-Ouchi, S Tamura, Y Tanaka and H Ishitsuka**

Cytostatics Research Group, Nippon Roche Research Center, 200 Kajiwara, Kamakura, Kanagawa 247-8530, Japan

**Summary** Murine colon 26 carcinoma growing at either subcutaneous (s.c.) or intramuscular (i.m.) inoculation sites causes cachexia in mice. Such animals show extensive loss of body weight, wasting of the muscle and adipose tissues, hypoglycaemia, and hypercalcaemia, even when the tumour weight comprises only about 1.9% of carcass weight. In contrast, the same tumour when inoculated into the liver does not cause any sign of tumour-related cachexia even when the tumour becomes much larger (6.6% of carcass weight). Interleukin 6 (IL-6), a mediator associated with cachexia in this tumour model, is detected at high levels both in the tumour tissues and in the circulating blood of mice bearing colon 26 tumour at the s.c. inoculation site. In contrast, only minute levels of IL-6 are detected in the tumour grown in the liver. The colon 26 tumour grown in the liver does not lose its ability to cause cachexia, because the tumour when re-inoculated s.c. is able to cause extensive weight loss and produce IL-6 as did the original colon 26 cell line. Histological studies revealed differences in the composition of tumour tissues: the tumours grown in the subcutis consist of many polygonal tumour cells, extended-intercellular space, and high vascular density, whereas those grown in the liver consist of spindle-shaped tumour cells. Thus, the environment where tumour cells grow would be a critical factor in determining the cachectic phenotype of cancer cells, including their ability to produce IL-6.

**Keywords:** colon 26; cachexia; IL-6; inoculation site; malignancy

We previously reported that, when the colon 26 murine colon carcinoma is inoculated into mice subcutaneously (s.c.), it causes progressive body weight loss, adipose tissue and muscle wasting, and other homeostasis disorders associated with cachexia (Tanaka et al, 1990). Cachexia is known to be caused by mediators produced by tumours or by the host as a result of tumour–host interaction. Previously, various factors have been proposed as cachexia mediators, such as tumour necrosis factor alpha (TNF-α) (Oliff et al, 1987; Tracey et al, 1988; Stovroff et al, 1988; Yoneda et al, 1991), interferon gamma (IFN-γ) (Matthys et al, 1991*a*, 1991*b*), interleukin (IL)-1α (Moldawer et al, 1987; Gelin et al, 1991), leukaemia inhibitory factor (LIF) (Mori et al, 1991) and IL-6 (Yoneda et al, 1993; Tamura et al, 1995). Fujimoto-Ouchi et al (1995) and Strassmann et al (1992*a*) reported that anti-IL-6 antibody suppressed the progressive body weight loss and other disorders associated with cachexia in mice bearing colon 26. It is therefore likely that IL-6 would be one of the cachexia mediators in the colon 26 model.

During our studies on cachexia in the colon 26 model, we discovered that tumour cells inoculated into the liver lost the ability to induce progressive weight loss, even though the same tumour cells inoculated into the subcutis or the muscle cause cachexia. Cancer cells often cause phenotypic changes even in vivo that are dependent upon the environment in which these cells are growing. Cachexia may be one such phenotype, the expression of which is dependent on that particular environment. The present study demonstrates that different cachexia phenotypes are produced by the same colon 26 cells when they are growing in different environments, subcutaneously (s.c.) and in the liver. In addition, we investigated such aspects of the tumour grown in the different environments as its IL-6 productivity and histological characteristics. Results from the present study suggest that cachexia in mice bearing colon 26 would be induced only in an environment where IL-6 is produced.

### MATERIALS AND METHODS

### Mice

Male 4-week-old BALB/c × DBA/2 F1 mice were obtained from SLC (Hamamatsu, Japan); they were used for experiments after being acclimated for 1 week. The animal house is kept at 24 ± 2°C with a 12-h dark–light cycle. The mice were fed with breeding diet F1 (Funabashi Farm, Funabashi, Japan), containing 21.3% protein, 57.1% carbohydrates, 5.6% fat, 3.3% fibre, 5.7% ash and 7.0% moisture (metabolic calories, 3.76 kcal/g), and given water ad libitum. All studies were conducted in accordance with ‘the Guidelines for the Care and Use of Laboratory Animals in Nippon Roche Research Center’.

### Tumour

A subclone of colon 26, clone 20 which causes progressive weight loss (Fujimoto-Ouchi et al, 1995), was used in the study. Colon 26 cells were maintained in vitro with Roswell Park Memorial Institute (RPMI)-1640 containing 10% fetal bovine serum in a humidified atmosphere with 5% carbon dioxide at 37°C. The cells were treated with 0.05% trypsin and 0.02% EDTA to obtain a single cell suspension and then inoculated into the liver ($1 \times 10^4$ cells) via a branch of the jejunal veins for one group of mice,

*Received 12 September 1997*
*Revised 29 July 1998*
*Accepted 4 August 1998*

*Correspondence to:* T Matsumoto

*Present address: AGENE Research Institute, 200 Kajiwara, Kamakura, Kanagawa 247-0063, Japan which were anaesthetized with pentobarbital sodium at 100 mg/kg intraperitoneally (i.p.). In the other group of mice, colon 26 cells ($5 \times 10^5$ cells) were inoculated into the subcutis of the right flank after a sham operation to reveal the jejunal vein. In some experiments, the colon 26 grown in the liver was cut into an approximately 8-$mm^3$ piece of tumour block and was re-transplanted s.c. into mice by means of a trocar. Another group of mice that were inoculated with colon 26 cells ($5 \times 10^5$ cells) into the right femoral rectus muscle was compared with a control group of mice injected with physiological saline into the same site.

### Measurement of body wasting

Body weight and tumour volume were measured once or twice a week. The volume of the tumour grown in the s.c. transplantation site was estimated by using the following equation, $ab^2/2$, where $a$ and $b$ are tumour length and width, respectively. The tumour weight grown in the liver was estimated by subtracting the average weight of the tumour-free livers of sham-operated mice from that of the tumour-transplanted liver. The carcass weight, the difference in weight between the whole body and tumour tissue, was also calculated. To determine the extent of the adipose tissue wasting, we measured the weight of the left epididymal adipose tissues.

### Assays

Blood samples were collected from the heart of chloroform-anaesthetized mice via cardiac puncture. Tumour tissues and the liver were homogenized in phosphate buffered saline (pH 7.4) on ice with a potter homogenizer and then centrifuged at 5000 ***g*** for 10 min. To determine the concentration of substances in serum or plasma and tissues to be tested, the following methods and reagents were used: an enzyme reaction method with mutarotase and glucose oxidase for measuring glucose by the Glucose CII test (Wako, Osaka, Japan); a colour-chelate method with *o*-cresol phthalein complexone for measuring calcium by the Calcium C-Test (Wako, Osaka, Japan) (Gitelman, 1967); enzyme-linked immunosorbent assay (ELISA) systems for measuring IL-6, TNF-α (Endogen, Boston, MA, USA) and IL-1α (Genzyme, Boston, MA, USA).

### Northern blotting

RNA was extracted from tumour tissues with TRIZOL reagent (GIBCO BRL, Life Technologies, Inc., Rockville, MD, USA). Twenty micrograms of total RNA were electrophoresed in 1% agarose gels containing formaldehyde and were transferred to Hybond-$N^+$ membranes (Amersham International plc, Buckinghamshire, UK). Hybridization was performed at 42°C for 16 h with a $2 \times 10^6$ cpm/ml $^{32}$P-radiolabelled probe prepared from the C-terminal region, which includes the 3´-untranslated region (nucleotide residues 532–992) of the mouse IL-6 cDNA. Then the membrane was washed in 2 × SSC/0.1% sodium dodecyl sulphate at 42°C for 2 h. Autoradiograms of the membrane were obtained using a BAS-1500 Bioimaging Analyzer (Fuji Film, Japan).

### Histology

Tissue specimens were obtained from tumours grown in the subcutis and in the liver. The tumour tissues were cut into thin slices and immersed in a Tissue-Tek OCT, for 20 min. The tumour slices were then frozen on dry ice, embedding in Tissue-Tek OCT and kept frozen at –80°C. Other slices of tumour tissues were fixed immediately with 10% neutral-buffered formalin in phosphate-buffered saline (PBS). The fixed tissues were dehydrated sequentially in ethanol and xylene, and then embedded in paraffin blocks. For haematoxylin–eosin staining, 2-μm-thick paraffin sections of tissues were placed on slides, deparaffinized with xylene, washed with ethanol, and then transferred to distilled water. The hydrated sections were then stained with haematoxylin and eosin solutions.

For immunohistochemical staining, 4-μm cryostat sections of the frozen embedded tissues were placed on slides and fixed for 10 min in 4°C acetone and then transferred to 0.1 M PBS (pH 7.4). The fixed slides were immunostained according to the ABC procedure (Hsu et al, 1981). Briefly, the slides were incubated with 3% skim milk containing PBS for 30 min at room temperature, with the rat monoclonal antibody to murine IL-6 (MM-600C, Endogen, MA, USA) overnight at 4°C, and with biotinylated rabbit anti-rat IgG antibody (BA-4001, Vector Laboratories, CA, USA) for 30 min at room temperature. This was followed by incubation in avidin–biotinylated peroxidase (PK-6100, Vector Laboratories, CA, USA) for 30 min at room temperature, and then they were developed in a mixture of diaminobenzidine and hydrogen peroxide for 5 min at room temperature, which resulted in a brown reaction product.

### Statistics

Differences in tumour size, tissue weight and concentrations of substances were compared by using the Mann–Whitney *U*-test. Differences were considered to be significant when the probability value was less than 0.05 ($P < 0.05$).

### RESULTS

### Tumour-inoculation site-dependent cachexia expression

Mice bearing colon 26 at the s.c. inoculation site lost body weight starting around day 10 after tumour inoculation (Figure 1). They were also partially immobile, and experienced asthenia and piloerection. On day 17, even when the tumour size was only 0.36 g, which was about 1.9% of carcass weight, significant decreases in carcass weight and adipose tissue, hypoglycaemia, and hypercalcaemia were observed (Table 1). The difference in the carcass weight between the tumour-bearing mice and the age-matched controls reached 8.7 g, and the adipose tissue weight of the cachectic mice was 69 ± 43 mg compared with 367 ± 41 mg for the control mice. In contrast, mice bearing the same colon 26 inoculated into the liver did not show any significant change in carcass weight and adipose tissue weight on day 17, even though the tumour weight was 1.61 g, which was about 6.6% of carcass weight. Nevertheless, hypoglycaemia and mild hypercalcaemia were observed (Table 1). The colon 26 grown in the liver (up to day 17) was removed, cut into small pieces, and re-inoculated s.c. with a trocar to investigate whether it had lost its ability to cause cachexia. Figure 2 and Table 1 show that its ability to cause cachexia remained intact and was the same as that of the original colon 26. Its growth pattern was also the same and it caused progressive weight loss starting around day 10 after tumour inoculation (Figure 2). It also caused substantial adipose tissue weight

© Cancer Research Campaign 1999

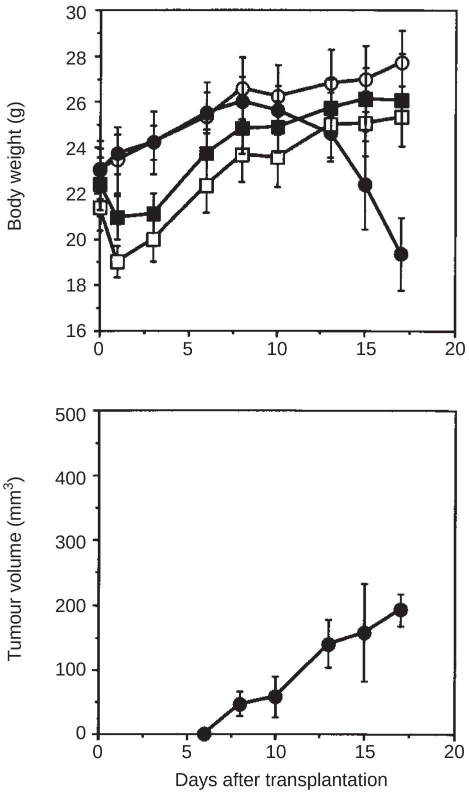

**Figure 1** Time-courses of changes in tumour volume and body weight of mice bearing colon 26. Groups of seven mice were inoculated on day 0 with colon 26 cells subcutaneously (●) or in the liver (■). Body weight and tumour volume were measured periodically. Body weight of age- and sex-matched control mice were monitored; normal mice (*n* = 6; ○) as the controls for those bearing the tumour in the subcutis and sham-operated mice for those bearing tumour in the liver (*n* = 6; □). Each value represents the mean ± SD

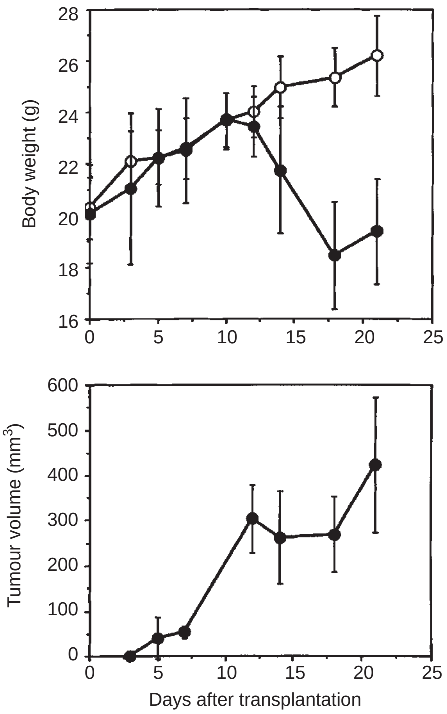

**Figure 2** Time-courses of changes in tumour volume and body weight of mice re-transplanted subcutaneously with colon 26 tumour grown in the liver. Seven mice were subcutaneously transplanted on day 0 with the tumour tissues (●). Body weight and tumour volume were measured periodically. Body weight of age- and sex-matched normal mice (*n* = 6) were also monitored (○). Each value represents the mean ± SD

loss even when the tumour size was small (3.3% of carcass weight) (Table 1).

Similar changes in carcass weight and adipose tissue weight were observed in mice bearing the colon 26 in their muscle (Table 1). In these mice, the tumour grew faster than did the one inoculated s.c. On day 13, when the mean tumour size was 0.67 g (3.5% of carcass weight), their carcass weight had decreased to 19.1 g, which was equivalent to 67% of that of the age-matched controls.

### Colon 26 causes cachexia producing IL-6

IL-6 is reported to be a cachexia mediator in the colon 26 model (Strassmann et al, 1992*a*; Fujimoto-Ouchi et al, 1995). We therefore investigated whether the colon 26 cells grown in the subcutis were producing IL-6. As Table 2 shows, a significant amount of IL-6 was detected in the colon 26 grown in the subcutis, whereas only slight amounts were found in the same tumour grown in the liver. In addition, higher serum IL-6 levels were detected in mice bearing the tumour grown in the subcutis than in the liver. The difference in the IL-6 expression levels was also observed when IL-6 mRNA levels in the colon 26 grown in the liver and in the subcutis were

**Table 1** Physiological changes observed in mice bearing the colon 26 carcinoma

| Inoculation site | Muscle | | Subcutis | | Liver | | Subcutis (retransplanted) | |
|---|---|---|---|---|---|---|---|---|
| Days after inoculation | 13 | | 17 | | 17 | | 21 | |
| Number of mice | Tumour-free | Tumour-bearing | Tumour-free | Tumour-bearing | Tumour-free | Tumour-bearing | Tumor-free | Tumor-bearing |
| | 5 | 4 | 6 | 7 | 7 | 7 | 6 | 7 |
| Parameters | | | | | | | | |
| Tumour weight (g) | – | 0.67 ± 0.16 | – | 0.36 ± 0.05 | – | 1.61 ± 0.85 | – | 0.59 ± 0.19 |
| Carcass weight (g) | 28.4 ± 1.0 | 19.1 ± 1.6[b] | 27.7 ± 1.4 | 19.0 ± 1.6[a] | 25.4 ± 1.3 | 24.5 ± 2.1 | 26.2 ± 1.6 | 17.8 ± 2.1[a] |
| Adipose tissue weight (mg) | 289 ± 30 | 31 ± 12[b] | 367 ± 41 | 69 ± 43[a] | 339 ± 70 | 315 ± 89 | 252 ± 60 | 33 ± 39[a] |
| Serum glucose (mg/dl) | 158 ± 25 | 63 ± 31[b] | 183 ± 22 | 73 ± 33[b] | 195 ± 27 | 100 ± 19[b] | 178 ± 8 | 103 ± 3[b] |
| Serum calcium (mg/dl) | 7.5 ± 0.9 | 17.8 ± 3.2[b] | 8.6 ± 1.1 | 15.5 ± 0.7[b] | 8.7 ± 1.3 | 11.2 ± 4.1 | 8.3 ± 0.7 | 13.0 ± 0.7[b] |

Colon 26 cells ($5 \times 10^5$) or pieces of colon 26 tumour tissue grown in the liver were inoculated into the subcutis. The mice were killed on the days indicated in the Table, and tumours and epididymal adipose tissues were weighted. Carcass weight was calculated by subtracting the tumour weight from the whole body weight. Each value represents the mean ± SD. [a], [b]significantly different from the value obtained in tumour-free mice ($P < 0.05, 0.01$).

 © Cancer Research Campaign 1999

**Table 2** Cytokine levels in mice bearing the colon 26 carcinoma

| | | Tumour-bearing mice | | |
|---|---|---|---|---|
| **Inoculation site** | **Tumour-free mice** | **Subcutis** | **Liver** | **Subcutis (retransplantation)** |
| **Number of mice** | **6** | **7** | **7** | **7** |
| Serum IL-6 (ng/ml) | < 0.02 | 1.64 ± 0.71 | 0.26 ± 0.31[a,b] | 1.03 ± 0.63 |
| IL-6 in the tumour tissue (ng/g) | – | 147 ± 69 | 8.8 ± 5.1[b] | 142 ± 119 |
| IL-6 in the liver tissue (ng/g) | 0.23 ± 0.06 | 0.17 ± 0.09 | NE | NE |
| IL-1α in the tumour tissue (ng/g) | – | 0.28 ± 0.22 | 0.82 ± 0.12 | 0.50 ± 0.44 |
| TNF-α in the tumour tissue (ng/g) | – | 31.2 ± 13.9 | 20.2 ± 3.5 | 16.1 ± 3.5 |

The levels of cytokines in sera and tissues from the same mice used in the experiment shown in Table 1 were determined by ELISA. Each value represents the mean ± SD. NE: Not examined, [a]IL-6 was not detected in three out of seven mice (< 0.02 ng/ml). [b]significantly different from the value obtained in mice bearing tumour subcutaneously ($P < 0.01$).

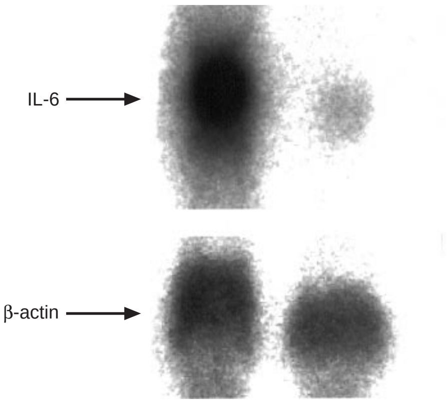

**Figure 3** Suppression of IL-6 mRNA expression in colon 26 tumour cells growing in the liver. Twenty micrograms of total RNAs, isolated from tumour tissue inoculated and grown in the subcutis or in the liver, were run on a denaturing agarose gel, and IL-6 mRNA was detected by Northern blot hybridization as a transcript of about 1.1 kb compared in a Northern blot analysis (Figure 3). The mRNA expression level was high in the tumour grown in the subcutis, whereas it was very low in that grown in the liver. Conversely, protein expression of the other cytokines so far tested in the tumour tissues, IL-1α and TNF-α, did not correlate with the cachexia expression, and these cytokines were not detected in the serum.

### Difference in histology of colon 26 grown in different inoculation sites

Figure 4 shows colon 26 tissues stained with haematoxylin and eosin solutions. The tumour tissues grown in the subcutis were characterized by many polygonal tumour cells resulting in extended intercellular space and condensed chromatin, and showing high vascular density. In contrast, histological characteristics of the same tumour grown in the liver differed from those of the s.c. tumour; all of the tumour cells were spindle-shaped with light nuclei and existed in close contact with one another. Figure 5 shows colon 26 tissues stained with anti-mouse IL-6 antibody. In the tumour tissues grown in the subcutis, most of the vital tumour cells were stained, particularly foci comprising several tumour cells; however, the tumour tissues grown in the liver were stained only very slightly (data not shown).

### DISCUSSION

Cachexia is one of the major obstacles in cancer therapy, and an appropriate treatment modality is necessary to counteract it.

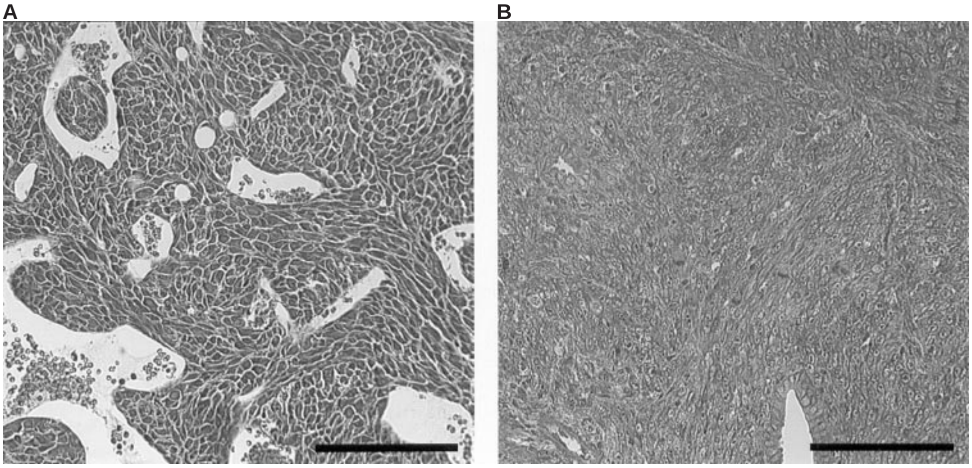

**Figure 4** Photomicrographs of sections of colon 26 tumour tissues. Formalin-fixed and paraffin-embedded tumour tissues were sectioned and stained with hematoxylin and eosin solution. (**A**) Colon 26 tumour tissue grown in the subcutis. (**B**) Colon 26 tumour tissue grown in the liver. Original magnification: × 100. Bars = 100 μm

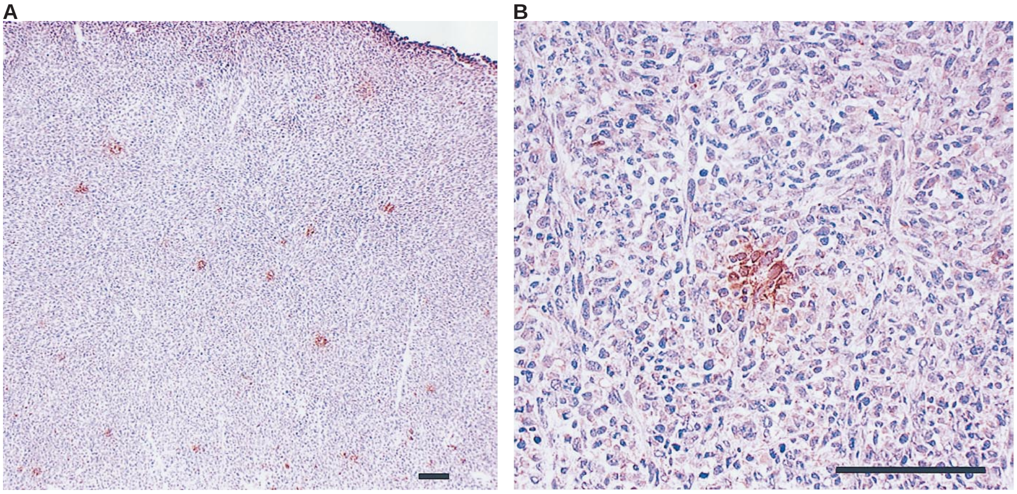

**Figure 5** Immuno-histochemical staining of colon 26 tumour tissue grown in the subcutis for IL-6. Tumour tissue sections were immunoperoxidase-stained with monoclonal antibody to murine IL-6. The sections were counterstained with haematoxylin. Original magnifications: (**A**) × 20; (**B**) × 100. Bar = 100 μm

However, the mechanisms that cause cachexia have not yet been fully investigated. We have shown that murine colon 26 carcinoma is an appropriate model for investigating the mechanisms that induce cachexia (Tanaka et al, 1990; Eda et al, 1991; Fujimoto-Ouchi et al, 1995). The present study demonstrated with this model that a cachexia-inducing phenotype is expressed when the tumour is growing in a particular tissue environment. The colon 26 inoculated s.c. causes extensive wasting of body weight and other abnormalities associated with cachexia even when the tumour size is small (Tanaka et al, 1990; Strassman et al, 1992*a*). The colon 26 grown in muscle was also able to cause cachexia. Interestingly, the same colon 26 did not cause body weight loss when inoculated into the liver. The colon 26 grown in the liver, however, again caused cachexia when it was removed and re-inoculated s.c.; this is similar to what was observed with the tumour when it was initially inoculated s.c. Because we used a freshly cloned cell line in the present study, the inoculation-site-dependent expression of cachexia would not be due to a clonal selection of the tumour cells losing their cachexia-inducing capability. The capability should be expressed by particular stimuli in the tumour environment.

Cachexia is known to be caused by mediators, and it has been reported that several cytokines, such as TNF-α, IL-1α, IL-6 and other factors, would be such mediators. Strassman et al (1992*a*) reported that an anti-IL-6 antibody protected mice from becoming cachectic in the colon 26 model; this would suggest that IL-6 is involved in cachexia induction. We confirmed the involvement of IL-6 in the colon 26 model, although it did not appear to be the sole mediator (Fujimoto-Ouchi et al, 1995). The present study demonstrated that the colon 26 grown in the subcutis expressed IL-6 mRNA and protein and caused cachexia, whereas the same tumour grown in the liver expressed IL-6 only slightly and did not cause cachexia. In contrast, neither IL-1α nor TNF-α production in the tumour correlated with the cachexia induction. Thus, colon 26 exposed to only particular stimuli in the subcutis would trigger IL-6 gene expression. The possibility, however, that an accelerated rate of IL-6 mRNA degradation in the liver results in no expression of cachexia phenotype cannot be excluded.

We do not yet know how IL-6 gene expression and protein levels are regulated in the colon 26 carcinoma. Factors in the tumour tissues may modulate the IL-6 expression. TNF-α and IL-1α are reported to up-regulate IL-6 production (Fiers, 1991; Strassmann et al, 1992*b*, 1993). It is not likely that these factors alone regulate the IL-6 expression in the colon 26, however, because levels of these factors did not correlate with cachexia induction. Steinkassere et al (1992) reported that a secreted form of IL-1 receptor antagonist, which suppresses IL-1 production (Arend et al, 1985), is expressed in the human liver. Therefore, an IL-1α receptor antagonist produced and secreted by host cells surrounding the tumour cells may down-regulate IL-6 production in the colon 26 growing in the liver. In fact, Strassmann et al (1993) reported that intratumoral injection of IL-1 receptor antagonist alleviated colon 26-induced cachexia.

Phenotypic expression of tumours is often affected by environmental factors. Productions of lectins in colon 26 (Vidal-Vanaclocha et al, 1991) and matrix metaloproteinase in colon cancer xenografts (Nakajima et al, 1990) were reported to be tumour inoculation-site-dependent. The present study showed that cachexia expression in colon 26 was indeed such a case. Differences in tumour environments may lead to the different phenotypic expressions, such as ability of colon 26 to cause tumour cachexia in the subcutis as well as in the muscle, but not in the liver. Factors that modulate expression of cachectic phenotypes of colon 26 should exist to a different extent between the tumours grown in the subcutis and in the liver. This is likely since the histological characteristics of colon 26 grown in the different inoculation sites are quite different. There were dispersed foci of tumour cells expressing high IL-6 immunoreactivity only in the tumour tissue growing in the subcutis. In addition, host cells, including macrophage-like cells, were infiltrated into the tumour. In contrast, fibroblast-like cells existed in the tumour grown in the liver. Strassmann et al (1992*b*) suggested that host macrophages interacting with colon 26 tumour cells produce IL-1, which in turn up-regulates the production of IL-6 in the tumour cells. The mechanism causing cachexia by colon 26 tumours in the particular environment remains to be clarified.

 © Cancer Research Campaign 1999

### REFERENCES

Arend WP, Joslin FG and Massoni RJ (1985) Effects of immune complexes on production by human monocytes of interleukin 1 or an interleukin 1 inhibitor. *J Immunol* **134**: 3868–3875

Eda H, Tanaka Y and Ishitsuka H (1991) 5´-Deoxy-5-fluorouridine improves cachexia by a mechanism independent of its antiproliferative action in colon 26 adenocarcinoma-bearing mice. *Cancer Chemother Pharmacol* **29**: 1–6

Fiers W (1991) Tumour necrosis factor: characterization at the molecular, cellular and in vivo level. *FEBS Lett* **285**: 199–212

Fujimoto-Ouchi K, Tamura S, Mori K, Tanaka Y and Ishitsuka H (1995) Establishment and characterization of cachexia inducing and non-inducing clones of murine colon 26 carcinoma. *Int J Cancer* **61**: 522–528

Gelin J, Moldawer LL, Lonnroth C, Sherry B, Chizonite R and Lundholm K (1991) Role of endogenous tumour necrosis factor a and interleukin 1 for experimental tumour growth and the development of cancer cachexia. *Cancer Res* **51**: 415–421

Gitelman HJ (1967) An improved automated procedure for the determination of calcium in biological specimens. *Anal Biochem* **18**: 521–531

Hsu SM, Raine L and Fanger H (1981) Use of avidin-biotin-peroxidase technique: a comparison between ABC and unlabeled antibody (PAP) procedures. *J Histochem Cytochem* **29**: 577–580

Matthys P, Dijkmans R, Proost P, Van Damme J, Heremans H, Sobis H and Billiau A (1991*a*) Severe cachexia in mice inoculated with interferon-γ-producing tumour cells. *Int J Cancer* **49**: 77–82

Matthys P, Heremans H, Opdenakker G and Billiau A (1991*b*) Anti-interferon-γ antibody treatment, growth of Lewis lung tumours in mice and tumour-associated cachexia. *Eur J Cancer* **27**: 182–187

Moldawer LL, Georgieff M and Lundholm K (1987) Interleukin1, tumour necrosis factor-α (cachectin) and the pathogenesis of cancer cachexia. *Clin Physiol* **7**: 263–274

Mori M, Yamaguchi K, Honda S, Nagasaki K, Ueda M, Abe O and Abe K (1991) Cancer cachexia syndrome developed in nude mice bearing melanoma cells producing leukemia-inhibitory factor. *Cancer Res* **51**: 6656–6659

Nakajima M, Morikawa K, Fabra A, Bucana CD and Fidler IJ (1990) Influence of organ environment on extracellular matrix degradative activity and metastasis of human colon carcinoma cells. *J Natl Cancer Inst* **82**: 1890–1898

Oliff A, Defeo-Jones D, Boyer M, Martinez D, Kiefer D, Vuocolo G, Wolfe A and Socher SH (1987) Tumour secreting human TNF/cachectin induce cachexia in mice. *Cell* **50**: 555–563

Steinkasserer A, Estaller C, Weiss EH and Sim RB (1992) Human interleukin-1 receptor antagonist is expressed in liver. *FEBS Lett* **310**: 60–62

Stovroff MC, Fraker DL, Swedenborg JA and Norton JA (1988) Cachectin/tumour necrosis factor-α possible mediator of cancer cachexia in the rat. *Cancer Res* **48**: 4567–4572

Strassmann G, Fong M, Kenney JS and Jacob CO (1992*a*) Evidence for the involvement of interleukin 6 in experimental cancer cachexia. *J Clin Invest* **89**: 1681–1684

Strassmann G, Jacob CO, Evans R, Beall D and Fong M (1992*b*) Mechanisms of experimental cachexia. Interaction between mononuclear phagocytes and colon-26 carcinoma and its relevance to IL-6-mediated cancer cachexia. *J Immunol* **148**: 3674–3678

Strassmann G, Masui Y, Chizzonite R and Fong M (1993) Mechanisms of experimental cancer cachexia. Local involvement of IL-1 in colon-26 tumour. *J Immunol* **150**: 2341–2345

Tamura S, Fujimoto K, Mori K, Endo M, Matsumoto T, Eda H, Tanaka Y, Ishitsuka H, Tokita H and Yamaguchi K (1995) Involvement of human interleukin 6 in experimental cachexia induced by a human uterine cervical carcinoma xenograft. *Clin Cancer Res* **1**: 1353–1358

Tanaka Y, Eda H, Tanaka T, Udagawa T, Ishikawa T, Horii I, Ishitsuka H, Kataoka T and Taguchi T (1990) Experimental cancer cachexia induced by transplantable colon 26 adenocarcinoma in mice. *Cancer Res* **50**: 2290–2295

Tracey KJ, Wei H, Manogue KR, Fong Y, Hesse DG, Nguyen HT, Kuo GC, Beutler B, Cotran RS, Cerami A and Lowry SF (1988) Cachectin/tumour necrosis factor induces cachexia, anemia, and inflammation. *J Exp Med* **167**: 1211–1227

Vidal-Vanaclocha F, Glaves D, Barbera-Guillem E and Weiss L (1991) Quantitative microscopy of mouse colon 26 cells growing in different metastatic sites. *Br J Cancer* **63**: 748–752

Yoneda T, Alsina MA, Chavez JB, Bonewald L, Nishimura R and Mundy GR (1991) Evidence that tumour necrosis factor plays a pathogenetic role in the paraneoplastic syndromes of cachexia, hypercalcemia, and leukocytosis in a human tumour in nude mice. *J Clin Invest* **87**: 977–985

Yoneda T, Nakai M, Moriyama K, Scott L, Ida N, Kunitomo T and Mundy GR (1993) Neutralizing antibodies to human interleukin 6 reverse hypercalcemia associated with a human squamous carcinoma. *Cancer Res* **53**: 737–740

*© Cancer Research Campaign 1999* 
